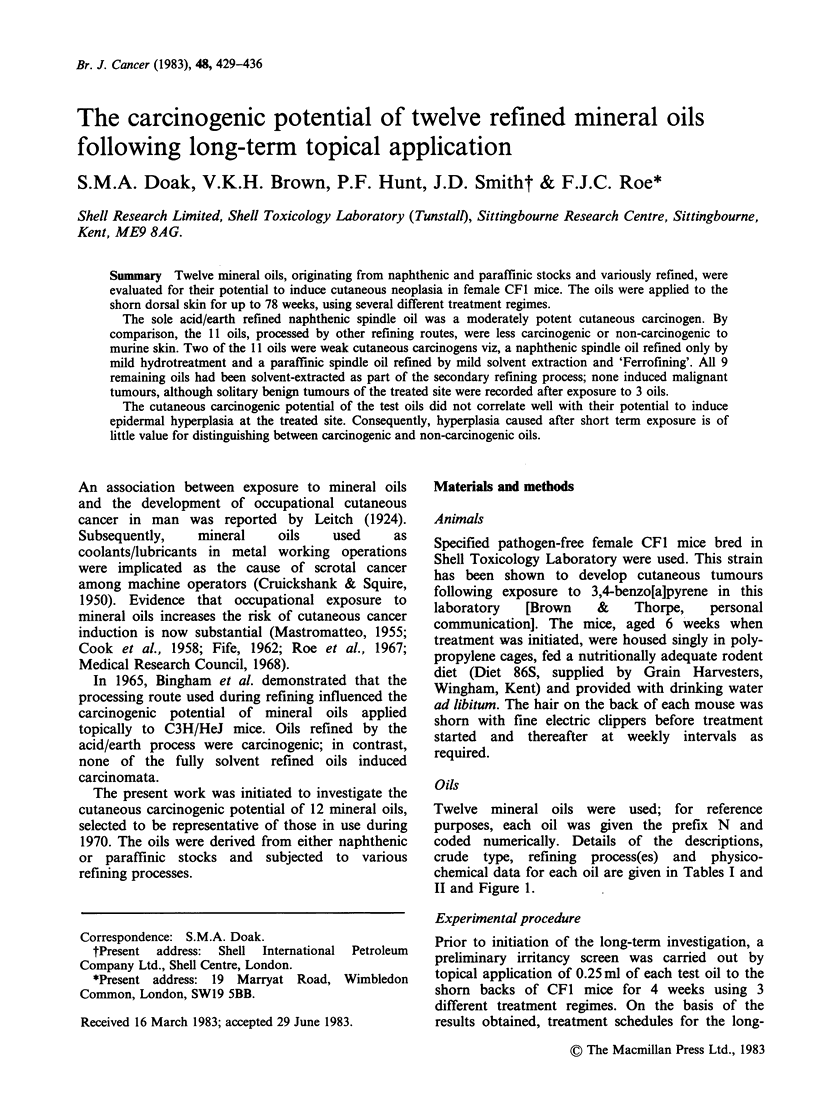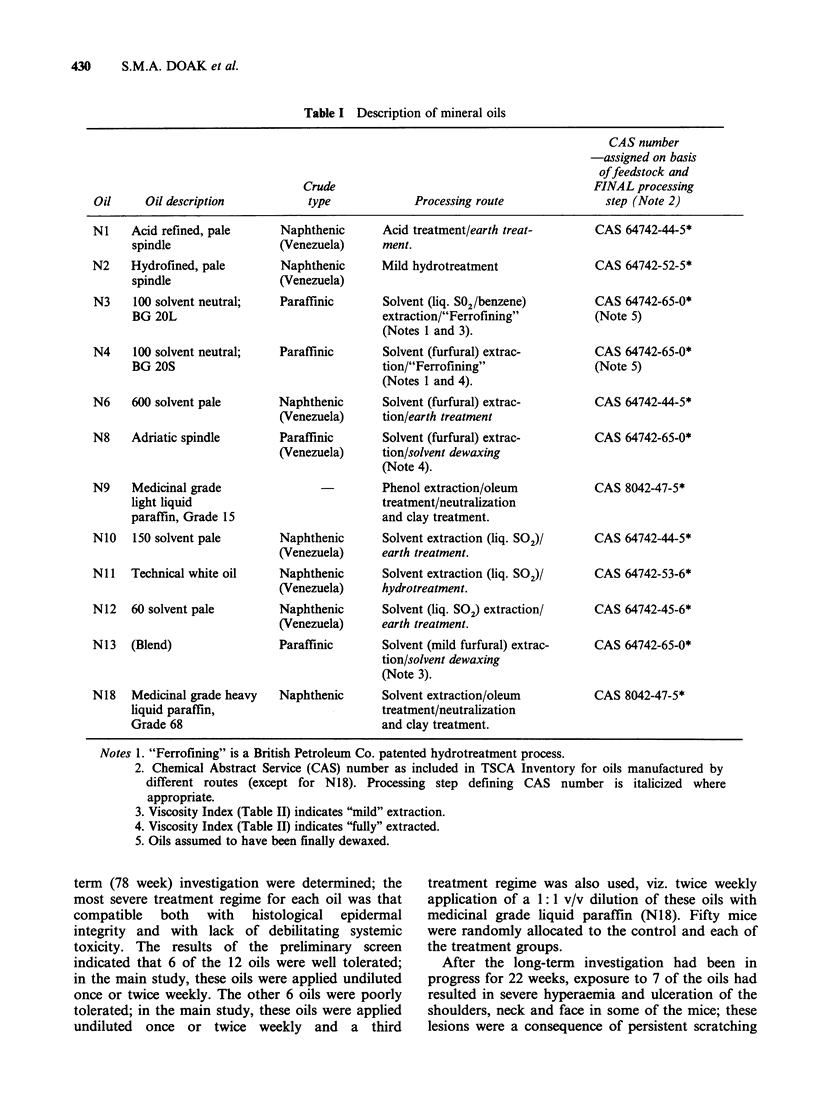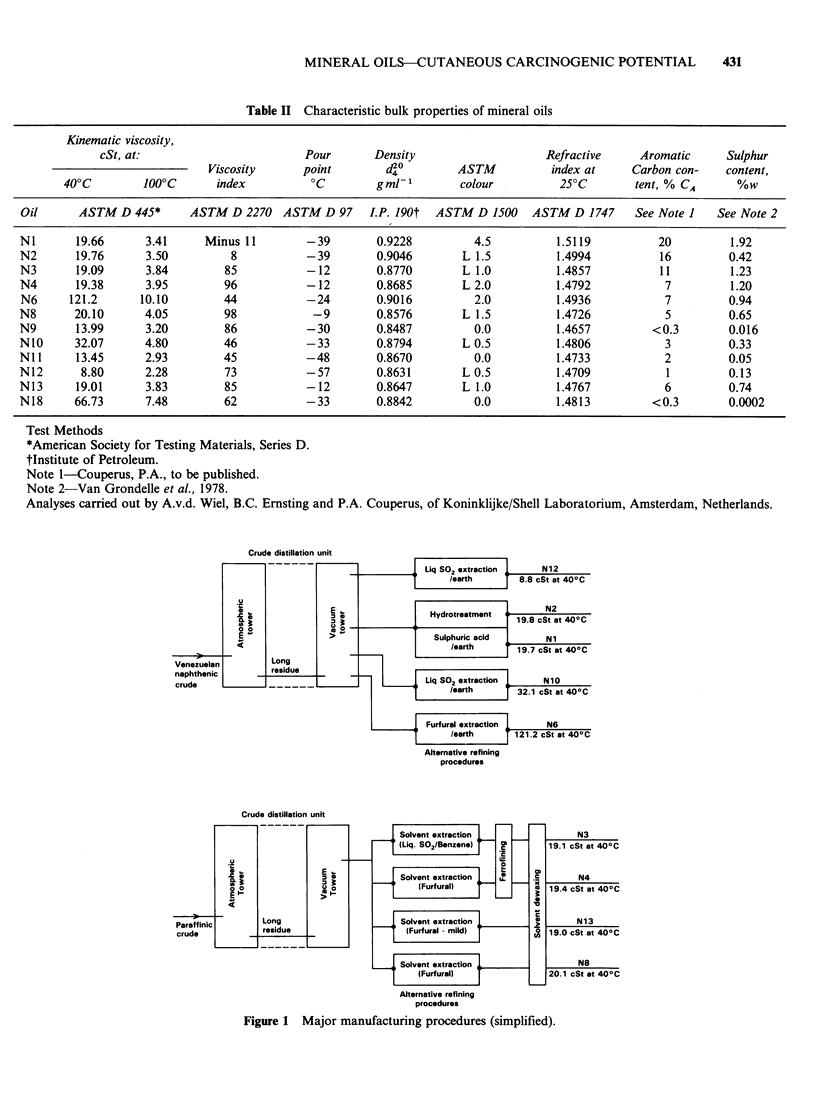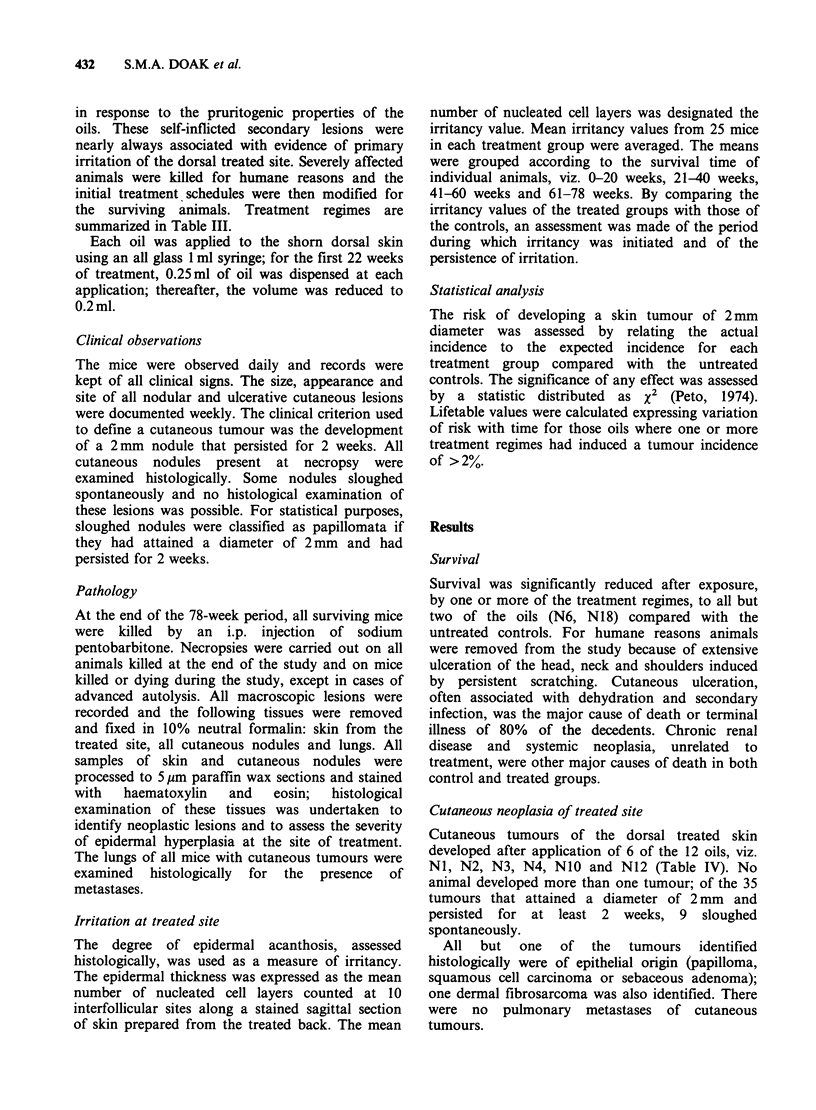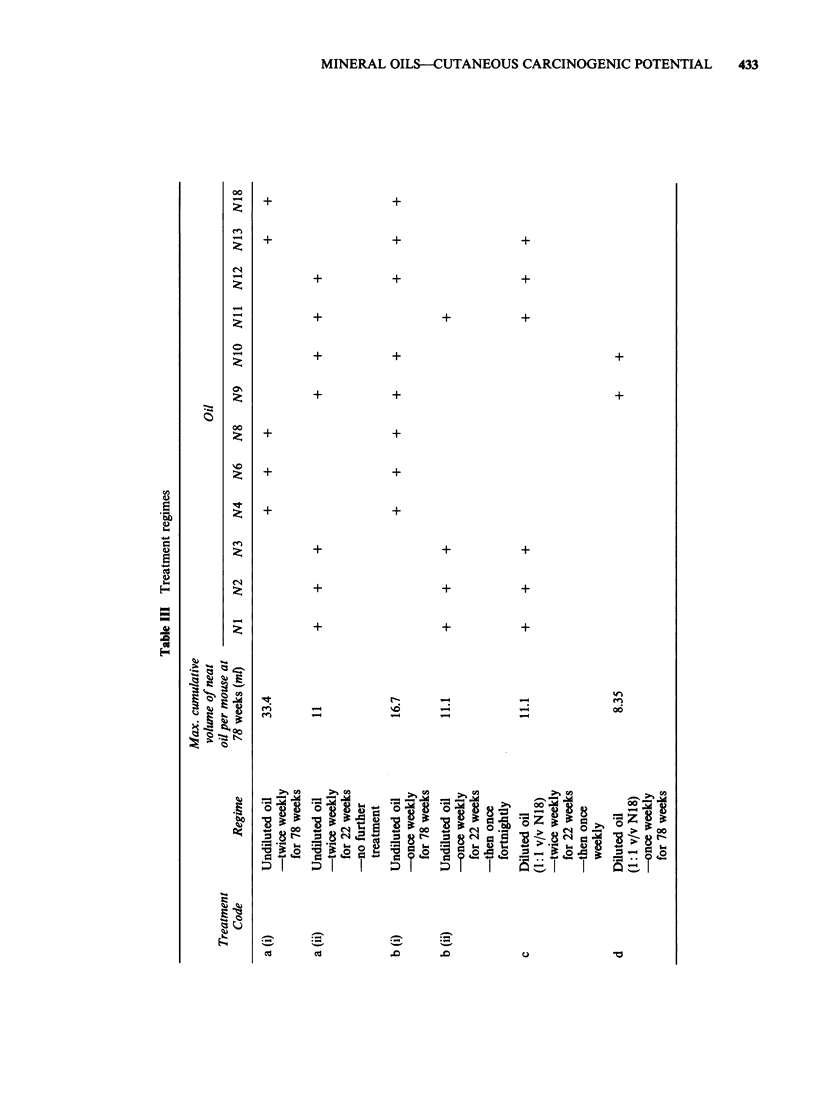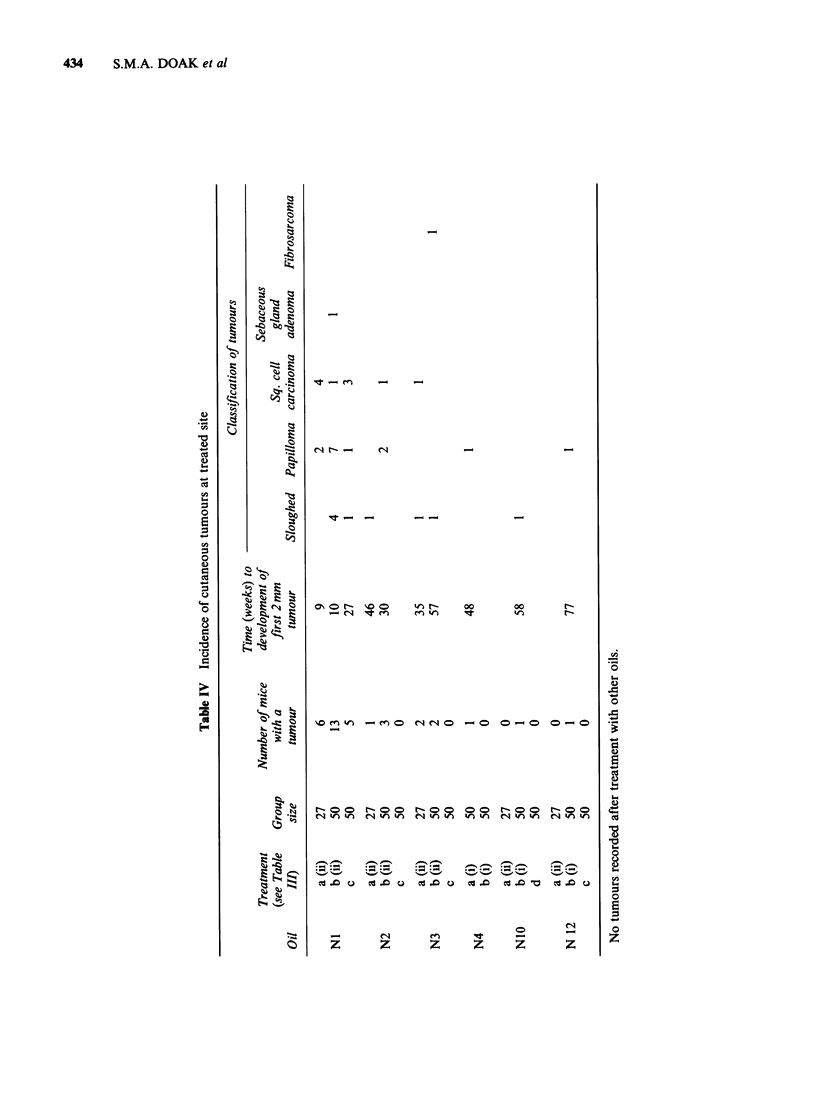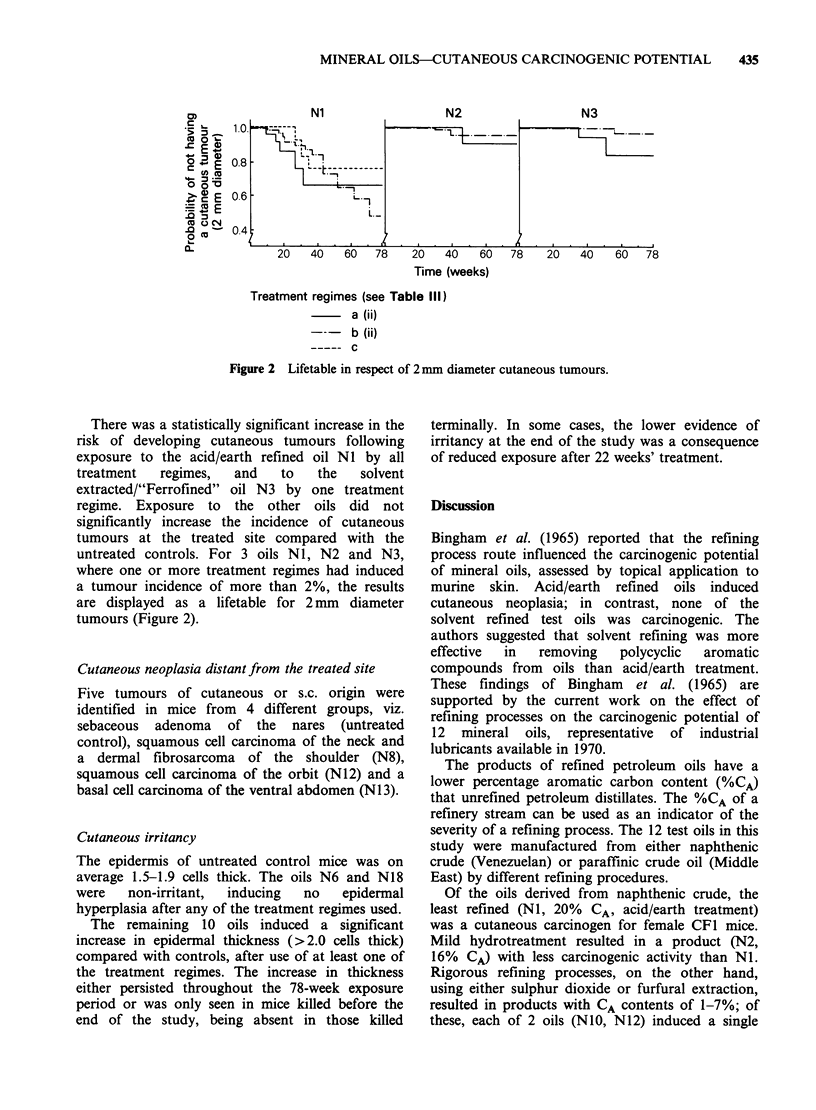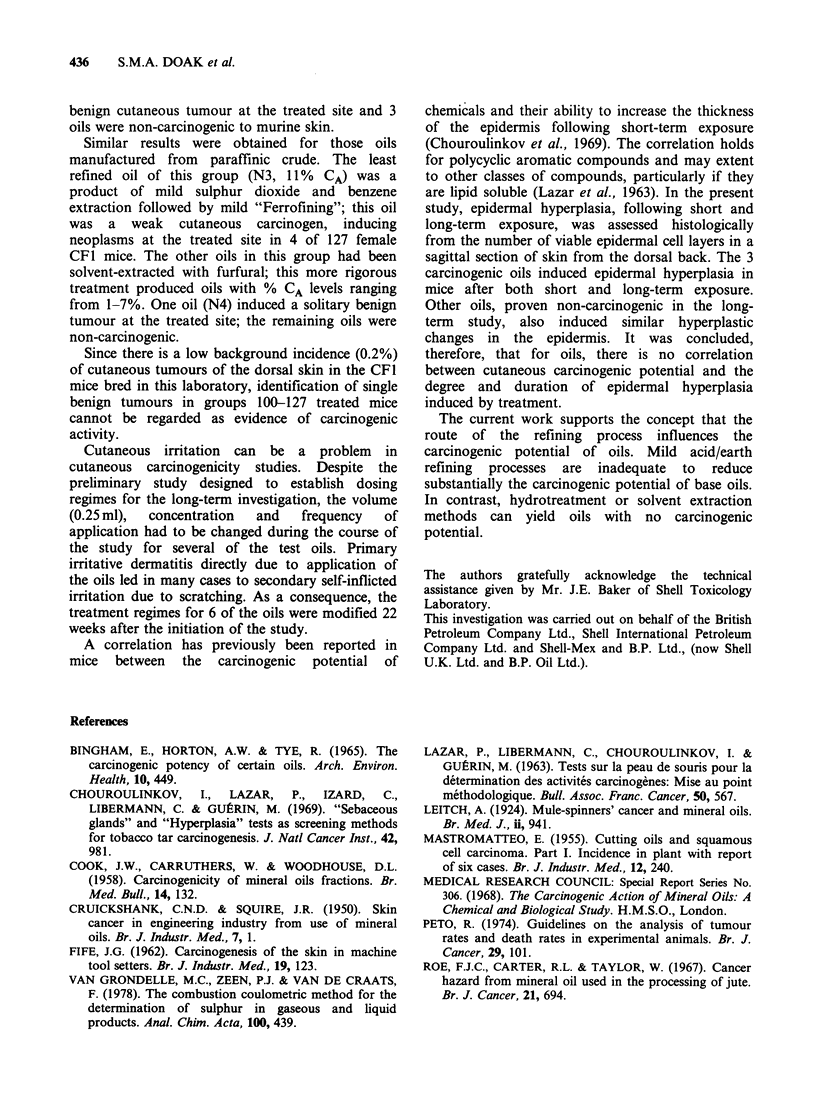# The carcinogenic potential of twelve refined mineral oils following long-term topical application.

**DOI:** 10.1038/bjc.1983.209

**Published:** 1983-09

**Authors:** S. M. Doak, V. K. Brown, P. F. Hunt, J. D. Smith, F. J. Roe

## Abstract

Twelve mineral oils, originating from naphthenic and paraffinic stocks and variously refined, were evaluated for their potential to induce cutaneous neoplasia in female CF1 mice. The oils were applied to the shorn dorsal skin for up to 78 weeks, using several different treatment regimes. The sole acid/earth refined naphthenic spindle oil was a moderately potent cutaneous carcinogen. By comparison, the 11 oils, processed by other refining routes, were less carcinogenic or non-carcinogenic to murine skin. Two of the 11 oils were weak cutaneous carcinogens viz, a naphthenic spindle oil refined only by mild hydrotreatment and a paraffinic spindle oil refined by mild solvent extraction and 'Ferrofining'. All 9 remaining oils had been solvent-extracted as part of the secondary refining process; none induced malignant tumours, although solitary benign tumours of the treated site were recorded after exposure to 3 oils. The cutaneous carcinogenic potential of the test oils did not correlate well with their potential to induce epidermal hyperplasia at the treated site. Consequently, hyperplasia caused after short term exposure is of little value for distinguishing between carcinogenic and non-carcinogenic oils.


*Br. J. Cancer* (1983), **48**, 429–436

### The carcinogenic potential of twelve refined mineral oils following long-term topical application

S.M.A. Doak, V.K.H. Brown, P.F. Hunt, J.D. Smith† & F.J.C. Roe*

*Shell Research Limited, Shell Toxicology Laboratory (Tunstall), Sittingbourne Research Centre, Sittingbourne, Kent, ME9 8AG.*

**Summary** Twelve mineral oils, originating from naphthenic and paraffinic stocks and variously refined, were evaluated for their potential to induce cutaneous neoplasia in female CF1 mice. The oils were applied to the shorn dorsal skin for up to 78 weeks, using several different treatment regimes.

The sole acid/earth refined naphthenic spindle oil was a moderately potent cutaneous carcinogen. By comparison, the 11 oils, processed by other refining routes, were less carcinogenic or non-carcinogenic to murine skin. Two of the 11 oils were weak cutaneous carcinogens viz, a naphthenic spindle oil refined only by mild hydrotreatment and a paraffinic spindle oil refined by mild solvent extraction and 'Ferrofining'. All 9 remaining oils had been solvent-extracted as part of the secondary refining process; none induced malignant tumours, although solitary benign tumours of the treated site were recorded after exposure to 3 oils.

The cutaneous carcinogenic potential of the test oils did not correlate well with their potential to induce epidermal hyperplasia at the treated site. Consequently, hyperplasia caused after short term exposure is of little value for distinguishing between carcinogenic and non-carcinogenic oils.

An association between exposure to mineral oils and the development of occupational cutaneous cancer in man was reported by Leitch (1924). Subsequently, mineral oils used as coolants/lubricants in metal working operations were implicated as the cause of scrotal cancer among machine operators (Cruickshank & Squire, 1950). Evidence that occupational exposure to mineral oils increases the risk of cutaneous cancer induction is now substantial (Mastromatteo, 1955; Cook *et al.*, 1958; Fife, 1962; Roe *et al.*, 1967; Medical Research Council, 1968).

In 1965, Bingham *et al.* demonstrated that the processing route used during refining influenced the carcinogenic potential of mineral oils applied topically to C3H/HeJ mice. Oils refined by the acid/earth process were carcinogenic; in contrast, none of the fully solvent refined oils induced carcinomata.

The present work was initiated to investigate the cutaneous carcinogenic potential of 12 mineral oils, selected to be representative of those in use during 1970. The oils were derived from either naphthenic or paraffinic stocks and subjected to various refining processes.

Correspondence: S.M.A. Doak.
†Present address: Shell International Petroleum Company Ltd., Shell Centre, London.
*Present address: 19 Marryat Road, Wimbledon Common, London, SW19 5BB.

Received 16 March 1983; accepted 29 June 1983.

### Materials and methods

### *Animals*

Specified pathogen-free female CF1 mice bred in Shell Toxicology Laboratory were used. This strain has been shown to develop cutaneous tumours following exposure to 3,4-benzo[a]pyrene in this laboratory [Brown & Thorpe, personal communication]. The mice, aged 6 weeks when treatment was initiated, were housed singly in polypropylene cages, fed a nutritionally adequate rodent diet (Diet 86S, supplied by Grain Harvesters, Wingham, Kent) and provided with drinking water *ad libitum*. The hair on the back of each mouse was shorn with fine electric clippers before treatment started and thereafter at weekly intervals as required.

### *Oils*

Twelve mineral oils were used; for reference purposes, each oil was given the prefix N and coded numerically. Details of the descriptions, crude type, refining process(es) and physicochemical data for each oil are given in Tables I and II and Figure 1.

### *Experimental procedure*

Prior to initiation of the long-term investigation, a preliminary irritancy screen was carried out by topical application of 0.25 ml of each test oil to the shorn backs of CF1 mice for 4 weeks using 3 different treatment regimes. On the basis of the results obtained, treatment schedules for the long-

© The Macmillan Press Ltd., 1983

**Table I** Description of mineral oils

| *Oil* | *Oil description* | *Crude type* | *Processing route* | *CAS number —assigned on basis of feedstock and FINAL processing step (Note 2)* |
|---|---|---|---|---|
| N1 | Acid refined, pale spindle | Naphthenic (Venezuela) | Acid treatment/*earth treatment.* | CAS 64742-44-5* |
| N2 | Hydrofined, pale spindle | Naphthenic (Venezuela) | Mild hydrotreatment | CAS 64742-52-5* |
| N3 | 100 solvent neutral; BG 20L | Paraffinic | Solvent (liq. $S0_2$/benzene) extraction/"Ferrofining" (Notes 1 and 3). | CAS 64742-65-0* (Note 5) |
| N4 | 100 solvent neutral; BG 20S | Paraffinic | Solvent (furfural) extraction/"Ferrofining" (Notes 1 and 4). | CAS 64742-65-0* (Note 5) |
| N6 | 600 solvent pale | Naphthenic (Venezuela) | Solvent (furfural) extraction/*earth treatment* | CAS 64742-44-5* |
| N8 | Adriatic spindle | Paraffinic (Venezuela) | Solvent (furfural) extraction/*solvent dewaxing* (Note 4). | CAS 64742-65-0* |
| N9 | Medicinal grade light liquid paraffin, Grade 15 | — | Phenol extraction/oleum treatment/neutralization and clay treatment. | CAS 8042-47-5* |
| N10 | 150 solvent pale | Naphthenic (Venezuela) | Solvent extraction (liq. $SO_2$)/ *earth treatment.* | CAS 64742-44-5* |
| N11 | Technical white oil | Naphthenic (Venezuela) | Solvent extraction (liq. $SO_2$)/ *hydrotreatment.* | CAS 64742-53-6* |
| N12 | 60 solvent pale | Naphthenic (Venezuela) | Solvent (liq. $SO_2$) extraction/ *earth treatment.* | CAS 64742-45-6* |
| N13 | (Blend) | Paraffinic | Solvent (mild furfural) extraction/*solvent dewaxing* (Note 3). | CAS 64742-65-0* |
| N18 | Medicinal grade heavy liquid paraffin, Grade 68 | Naphthenic | Solvent extraction/oleum treatment/neutralization and clay treatment. | CAS 8042-47-5* |

*Notes* 1. "Ferrofining" is a British Petroleum Co. patented hydrotreatment process.
2. Chemical Abstract Service (CAS) number as included in TSCA Inventory for oils manufactured by different routes (except for N18). Processing step defining CAS number is italicized where appropriate.
3. Viscosity Index (Table II) indicates "mild" extraction.
4. Viscosity Index (Table II) indicates "fully" extracted.
5. Oils assumed to have been finally dewaxed.

term (78 week) investigation were determined; the most severe treatment regime for each oil was that compatible both with histological epidermal integrity and with lack of debilitating systemic toxicity. The results of the preliminary screen indicated that 6 of the 12 oils were well tolerated; in the main study, these oils were applied undiluted once or twice weekly. The other 6 oils were poorly tolerated; in the main study, these oils were applied undiluted once or twice weekly and a third treatment regime was also used, viz. twice weekly application of a 1:1 v/v dilution of these oils with medicinal grade liquid paraffin (N18). Fifty mice were randomly allocated to the control and each of the treatment groups.

After the long-term investigation had been in progress for 22 weeks, exposure to 7 of the oils had resulted in severe hyperaemia and ulceration of the shoulders, neck and face in some of the mice; these lesions were a consequence of persistent scratching

**Table II** Characteristic bulk properties of mineral oils

| | *Kinematic viscosity, cSt, at:* | | | | | | | | |
|---|---|---|---|---|---|---|---|---|---|
| | *40°C* | *100°C* | *Viscosity index* | *Pour point °C* | *Density* $d_4^{20}$ *g ml*$^{-1}$ | *ASTM colour* | *Refractive index at 25°C* | *Aromatic Carbon content, %* $C_A$ | *Sulphur content, %w* |
| *Oil* | *ASTM D 445** | | *ASTM D 2270* | *ASTM D 97* | *I.P. 190†* | *ASTM D 1500* | *ASTM D 1747* | *See Note 1* | *See Note 2* |
| N1 | 19.66 | 3.41 | Minus 11 | −39 | 0.9228 | 4.5 | 1.5119 | 20 | 1.92 |
| N2 | 19.76 | 3.50 | 8 | −39 | 0.9046 | L 1.5 | 1.4994 | 16 | 0.42 |
| N3 | 19.09 | 3.84 | 85 | −12 | 0.8770 | L 1.0 | 1.4857 | 11 | 1.23 |
| N4 | 19.38 | 3.95 | 96 | −12 | 0.8685 | L 2.0 | 1.4792 | 7 | 1.20 |
| N6 | 121.2 | 10.10 | 44 | −24 | 0.9016 | 2.0 | 1.4936 | 7 | 0.94 |
| N8 | 20.10 | 4.05 | 98 | −9 | 0.8576 | L 1.5 | 1.4726 | 5 | 0.65 |
| N9 | 13.99 | 3.20 | 86 | −30 | 0.8487 | 0.0 | 1.4657 | <0.3 | 0.016 |
| N10 | 32.07 | 4.80 | 46 | −33 | 0.8794 | L 0.5 | 1.4806 | 3 | 0.33 |
| N11 | 13.45 | 2.93 | 45 | −48 | 0.8670 | 0.0 | 1.4733 | 2 | 0.05 |
| N12 | 8.80 | 2.28 | 73 | −57 | 0.8631 | L 0.5 | 1.4709 | 1 | 0.13 |
| N13 | 19.01 | 3.83 | 85 | −12 | 0.8647 | L 1.0 | 1.4767 | 6 | 0.74 |
| N18 | 66.73 | 7.48 | 62 | −33 | 0.8842 | 0.0 | 1.4813 | <0.3 | 0.0002 |

Test Methods
*American Society for Testing Materials, Series D.
†Institute of Petroleum.
Note 1—Couperus, P.A., to be published.
Note 2—Van Grondelle *et al.*, 1978.
Analyses carried out by A.v.d. Wiel, B.C. Ernsting and P.A. Couperus, of Koninklijke/Shell Laboratorium, Amsterdam, Netherlands.

**Figure 1** Major manufacturing procedures (simplified).

in response to the pruritogenic properties of the oils. These self-inflicted secondary lesions were nearly always associated with evidence of primary irritation of the dorsal treated site. Severely affected animals were killed for humane reasons and the initial treatment schedules were then modified for the surviving animals. Treatment regimes are summarized in Table III.

Each oil was applied to the shorn dorsal skin using an all glass 1 ml syringe; for the first 22 weeks of treatment, 0.25 ml of oil was dispensed at each application; thereafter, the volume was reduced to 0.2 ml.

*Clinical observations*

The mice were observed daily and records were kept of all clinical signs. The size, appearance and site of all nodular and ulcerative cutaneous lesions were documented weekly. The clinical criterion used to define a cutaneous tumour was the development of a 2 mm nodule that persisted for 2 weeks. All cutaneous nodules present at necropsy were examined histologically. Some nodules sloughed spontaneously and no histological examination of these lesions was possible. For statistical purposes, sloughed nodules were classified as papillomata if they had attained a diameter of 2 mm and had persisted for 2 weeks.

*Pathology*

At the end of the 78-week period, all surviving mice were killed by an i.p. injection of sodium pentobarbitone. Necropsies were carried out on all animals killed at the end of the study and on mice killed or dying during the study, except in cases of advanced autolysis. All macroscopic lesions were recorded and the following tissues were removed and fixed in 10% neutral formalin: skin from the treated site, all cutaneous nodules and lungs. All samples of skin and cutaneous nodules were processed to 5 $\mu$m paraffin wax sections and stained with haematoxylin and eosin; histological examination of these tissues was undertaken to identify neoplastic lesions and to assess the severity of epidermal hyperplasia at the site of treatment. The lungs of all mice with cutaneous tumours were examined histologically for the presence of metastases.

*Irritation at treated site*

The degree of epidermal acanthosis, assessed histologically, was used as a measure of irritancy. The epidermal thickness was expressed as the mean number of nucleated cell layers counted at 10 interfollicular sites along a stained sagittal section of skin prepared from the treated back. The mean number of nucleated cell layers was designated the irritancy value. Mean irritancy values from 25 mice in each treatment group were averaged. The means were grouped according to the survival time of individual animals, viz. 0–20 weeks, 21–40 weeks, 41–60 weeks and 61–78 weeks. By comparing the irritancy values of the treated groups with those of the controls, an assessment was made of the period during which irritancy was initiated and of the persistence of irritation.

*Statistical analysis*

The risk of developing a skin tumour of 2 mm diameter was assessed by relating the actual incidence to the expected incidence for each treatment group compared with the untreated controls. The significance of any effect was assessed by a statistic distributed as $\chi^2$ (Peto, 1974). Lifetable values were calculated expressing variation of risk with time for those oils where one or more treatment regimes had induced a tumour incidence of $>2\%$.

### Results

*Survival*

Survival was significantly reduced after exposure, by one or more of the treatment regimes, to all but two of the oils (N6, N18) compared with the untreated controls. For humane reasons animals were removed from the study because of extensive ulceration of the head, neck and shoulders induced by persistent scratching. Cutaneous ulceration, often associated with dehydration and secondary infection, was the major cause of death or terminal illness of 80% of the decedents. Chronic renal disease and systemic neoplasia, unrelated to treatment, were other major causes of death in both control and treated groups.

*Cutaneous neoplasia of treated site*

Cutaneous tumours of the dorsal treated skin developed after application of 6 of the 12 oils, viz. N1, N2, N3, N4, N10 and N12 (Table IV). No animal developed more than one tumour; of the 35 tumours that attained a diameter of 2 mm and persisted for at least 2 weeks, 9 sloughed spontaneously.

All but one of the tumours identified histologically were of epithelial origin (papilloma, squamous cell carcinoma or sebaceous adenoma); one dermal fibrosarcoma was also identified. There were no pulmonary metastases of cutaneous tumours.

**Table III** Treatment regimes

| *Treatment Code* | *Regime* | *Max. cumulative volume of neat oil per mouse at 78 weeks (ml)* | *Oil* | | | | | | | | | | | |
|---|---|---|---|---|---|---|---|---|---|---|---|---|---|---|
| | | | *N1* | *N2* | *N3* | *N4* | *N6* | *N8* | *N9* | *N10* | *N11* | *N12* | *N13* | *N18* |
| a (i) | Undiluted oil —twice weekly for 78 weeks | 33.4 | | | | + | + | + | | | | | + | + |
| a (ii) | Undiluted oil —twice weekly for 22 weeks —no further treatment | 11 | + | + | + | | | | + | + | + | + | | |
| b (i) | Undiluted oil —once weekly for 78 weeks | 16.7 | | | | + | + | + | + | + | | + | + | + |
| b (ii) | Undiluted oil —once weekly for 22 weeks —then once fortnightly | 11.1 | + | + | + | | | | | | + | | | |
| c | Diluted oil (1:1 v/v N18) —twice weekly for 22 weeks —then once weekly | 11.1 | + | + | + | | | | | | + | + | + | |
| d | Diluted oil (1:1 v/v N18) —once weekly for 78 weeks | 8.35 | | | | | | | + | + | | | | |

**Table IV** Incidence of cutaneous tumours at treated site

| Oil | Treatment (see Table III) | Group size | Number of mice with a tumour | Time (weeks) to development of first 2 mm tumour | Classification of tumours: Sloughed | Papilloma | Sq. cell carcinoma | Sebaceous gland adenoma | Fibrosarcoma |
|---|---|---|---|---|---|---|---|---|---|
| N1 | a (ii) | 27 | 6 | 9 | | 2 | 4 | | |
| | b (ii) | 50 | 13 | 10 | 4 | 7 | 1 | 1 | |
| | c | 50 | 5 | 27 | 1 | 1 | 3 | | |
| N2 | a (ii) | 27 | 1 | 46 | 1 | | | | |
| | b (ii) | 50 | 3 | 30 | | 2 | 1 | | |
| | c | 50 | 0 | | | | | | |
| N3 | a (ii) | 27 | 2 | 35 | 1 | | 1 | | |
| | b (ii) | 50 | 2 | 57 | 1 | | | | 1 |
| | c | 50 | 0 | | | | | | |
| N4 | a (i) | 50 | 1 | 48 | | 1 | | | |
| | b (i) | 50 | 0 | | | | | | |
| N10 | a (ii) | 27 | 0 | | | | | | |
| | b (i) | 50 | 1 | 58 | 1 | | | | |
| | d | 50 | 0 | | | | | | |
| N 12 | a (ii) | 27 | 0 | | | | | | |
| | b (i) | 50 | 1 | 77 | | 1 | | | |
| | c | 50 | 0 | | | | | | |

No tumours recorded after treatment with other oils.

**Figure 2** Lifetable in respect of 2 mm diameter cutaneous tumours.

There was a statistically significant increase in the risk of developing cutaneous tumours following exposure to the acid/earth refined oil N1 by all treatment regimes, and to the solvent extracted/"Ferrofined" oil N3 by one treatment regime. Exposure to the other oils did not significantly increase the incidence of cutaneous tumours at the treated site compared with the untreated controls. For 3 oils N1, N2 and N3, where one or more treatment regimes had induced a tumour incidence of more than 2%, the results are displayed as a lifetable for 2 mm diameter tumours (Figure 2).

### *Cutaneous neoplasia distant from the treated site*

Five tumours of cutaneous or s.c. origin were identified in mice from 4 different groups, viz. sebaceous adenoma of the nares (untreated control), squamous cell carcinoma of the neck and a dermal fibrosarcoma of the shoulder (N8), squamous cell carcinoma of the orbit (N12) and a basal cell carcinoma of the ventral abdomen (N13).

### *Cutaneous irritancy*

The epidermis of untreated control mice was on average 1.5–1.9 cells thick. The oils N6 and N18 were non-irritant, inducing no epidermal hyperplasia after any of the treatment regimes used.

The remaining 10 oils induced a significant increase in epidermal thickness (>2.0 cells thick) compared with controls, after use of at least one of the treatment regimes. The increase in thickness either persisted throughout the 78-week exposure period or was only seen in mice killed before the end of the study, being absent in those killed terminally. In some cases, the lower evidence of irritancy at the end of the study was a consequence of reduced exposure after 22 weeks' treatment.

### Discussion

Bingham *et al.* (1965) reported that the refining process route influenced the carcinogenic potential of mineral oils, assessed by topical application to murine skin. Acid/earth refined oils induced cutaneous neoplasia; in contrast, none of the solvent refined test oils was carcinogenic. The authors suggested that solvent refining was more effective in removing polycyclic aromatic compounds from oils than acid/earth treatment. These findings of Bingham *et al.* (1965) are supported by the current work on the effect of refining processes on the carcinogenic potential of 12 mineral oils, representative of industrial lubricants available in 1970.

The products of refined petroleum oils have a lower percentage aromatic carbon content (%$C_A$) that unrefined petroleum distillates. The %$C_A$ of a refinery stream can be used as an indicator of the severity of a refining process. The 12 test oils in this study were manufactured from either naphthenic crude (Venezuelan) or paraffinic crude oil (Middle East) by different refining procedures.

Of the oils derived from naphthenic crude, the least refined (N1, 20% $C_A$, acid/earth treatment) was a cutaneous carcinogen for female CF1 mice. Mild hydrotreatment resulted in a product (N2, 16% $C_A$) with less carcinogenic activity than N1. Rigorous refining processes, on the other hand, using either sulphur dioxide or furfural extraction, resulted in products with $C_A$ contents of 1–7%; of these, each of 2 oils (N10, N12) induced a single benign cutaneous tumour at the treated site and 3 oils were non-carcinogenic to murine skin.

Similar results were obtained for those oils manufactured from paraffinic crude. The least refined oil of this group (N3, 11% $C_A$) was a product of mild sulphur dioxide and benzene extraction followed by mild "Ferrofining"; this oil was a weak cutaneous carcinogen, inducing neoplasms at the treated site in 4 of 127 female CF1 mice. The other oils in this group had been solvent-extracted with furfural; this more rigorous treatment produced oils with % $C_A$ levels ranging from 1–7%. One oil (N4) induced a solitary benign tumour at the treated site; the remaining oils were non-carcinogenic.

Since there is a low background incidence (0.2%) of cutaneous tumours of the dorsal skin in the CF1 mice bred in this laboratory, identification of single benign tumours in groups 100–127 treated mice cannot be regarded as evidence of carcinogenic activity.

Cutaneous irritation can be a problem in cutaneous carcinogenicity studies. Despite the preliminary study designed to establish dosing regimes for the long-term investigation, the volume (0.25 ml), concentration and frequency of application had to be changed during the course of the study for several of the test oils. Primary irritative dermatitis directly due to application of the oils led in many cases to secondary self-inflicted irritation due to scratching. As a consequence, the treatment regimes for 6 of the oils were modified 22 weeks after the initiation of the study.

A correlation has previously been reported in mice between the carcinogenic potential of chemicals and their ability to increase the thickness of the epidermis following short-term exposure (Chouroulinkov *et al.*, 1969). The correlation holds for polycyclic aromatic compounds and may extent to other classes of compounds, particularly if they are lipid soluble (Lazar *et al.*, 1963). In the present study, epidermal hyperplasia, following short and long-term exposure, was assessed histologically from the number of viable epidermal cell layers in a sagittal section of skin from the dorsal back. The 3 carcinogenic oils induced epidermal hyperplasia in mice after both short and long-term exposure. Other oils, proven non-carcinogenic in the long-term study, also induced similar hyperplastic changes in the epidermis. It was concluded, therefore, that for oils, there is no correlation between cutaneous carcinogenic potential and the degree and duration of epidermal hyperplasia induced by treatment.

The current work supports the concept that the route of the refining process influences the carcinogenic potential of oils. Mild acid/earth refining processes are inadequate to reduce substantially the carcinogenic potential of base oils. In contrast, hydrotreatment or solvent extraction methods can yield oils with no carcinogenic potential.

The authors gratefully acknowledge the technical assistance given by Mr. J.E. Baker of Shell Toxicology Laboratory.
This investigation was carried out on behalf of the British Petroleum Company Ltd., Shell International Petroleum Company Ltd. and Shell-Mex and B.P. Ltd., (now Shell U.K. Ltd. and B.P. Oil Ltd.).

### References

BINGHAM, E., HORTON, A.W. & TYE, R. (1965). The carcinogenic potency of certain oils. *Arch. Environ. Health*, **10**, 449.

CHOUROULINKOV, I., LAZAR, P., IZARD, C., LIBERMANN, C. & GUÉRIN, M. (1969). "Sebaceous glands" and "Hyperplasia" tests as screening methods for tobacco tar carcinogenesis. *J. Natl Cancer Inst.*, **42**, 981.

COOK, J.W., CARRUTHERS, W. & WOODHOUSE, D.L. (1958). Carcinogenicity of mineral oils fractions. *Br. Med. Bull.*, **14**, 132.

CRUICKSHANK, C.N.D. & SQUIRE, J.R. (1950). Skin cancer in engineering industry from use of mineral oils. *Br. J. Industr. Med.*, **7**, 1.

FIFE, J.G. (1962). Carcinogenesis of the skin in machine tool setters. *Br. J. Industr. Med.*, **19**, 123.

VAN GRONDELLE, M.C., ZEEN, P.J. & VAN DE CRAATS, F. (1978). The combustion coulometric method for the determination of sulphur in gaseous and liquid products. *Anal. Chim. Acta*, **100**, 439.

LAZAR, P., LIBERMANN, C., CHOUROULINKOV, I. & GUÉRIN, M. (1963). Tests sur la peau de souris pour la détermination des activités carcinogènes: Mise au point méthodologique. *Bull. Assoc. Franc. Cancer*, **50**, 567.

LEITCH, A. (1924). Mule-spinners' cancer and mineral oils. *Br. Med. J.*, **ii**, 941.

MASTROMATTEO, E. (1955). Cutting oils and squamous cell carcinoma. Part I. Incidence in plant with report of six cases. *Br. J. Industr. Med.*, **12**, 240.

MEDICAL RESEARCH COUNCIL: Special Report Series No. 306. (1968). *The Carcinogenic Action of Mineral Oils: A Chemical and Biological Study*. H.M.S.O., London.

PETO, R. (1974). Guidelines on the analysis of tumour rates and death rates in experimental animals. *Br. J. Cancer*, **29**, 101.

ROE, F.J.C., CARTER, R.L. & TAYLOR, W. (1967). Cancer hazard from mineral oil used in the processing of jute. *Br. J. Cancer*, **21**, 694.